# Hospital Benefit for Insured Persons by Pro Rata Payments and Not as Charity

**Published:** 1912-12-07

**Authors:** F. W. Francis

**Affiliations:** Chairman of Audit Committee, Warneford Hospital, Leamington, and Vice-Chairman Warwickshire Insurance Committee.


### The Editor's Letter-Box.

### Hospital Benefit for Insured Persons by Pro Rata Payments and not as Charity.

*To the Editor of* THE HOSPITAL.

SIR,—It is the charm of THE HOSPITAL that it prints in its columns letters from those who differ from its policy or controvert any particular statement: may I venture again to trespass on your courtesy? In your last issue it is asserted that institutional in-patient treatment has been paid for by insured persons and is State-guaranteed. Let us go to the Act itself and see if this is so. In Section 8 it is stated that the first benefit conferred by the Act is medical treatment and attendance, including the provision of proper and sufficient medicine and certain medical and surgical appliances. No word about institutional treatment in connection with medical benefit.

What is medical benefit? Refer to Section 15: (1) Every Insurance Committee shall for the purpose of administering medical benefit make arrangements with duly qualified medical practitioners; and in Section 15: (5) Every committee shall make arrangements for the supply of proper and sufficient drugs and medicines, etc. Not one word about making arrangements for institutional treatment. Insurance committees have no power under the Act to make such arrangements (except for tuberculosis in so far as it is treated as a sanatorium benefit); and I would emphatically repeat that they have not a single penny at their disposal for such a purpose.

It may be that it is desirable to pass another Act of Parliament compelling local authorities to support the hospitals and institutions required for institutional treatment of sick persons; but that is another story; insured persons have, according to the Act, no right to any medical benefit except treatment by practitioners on the panel.

I am, Sir,
Your obedient servant,
F. W. FRANCIS,
Chairman of Audit Committee, Warneford Hospital, Leamington, and Vice-Chairman, Warwickshire Insurance Committee.

December 2.

[We thank our correspondent for his letter. We always welcome criticism, correction and suggestions. We can find no statement in our last issue to the effect that "institutional in-patient treatment has been paid for by insured persons and is State-guaranteed." On the contrary we have from the first made it clear that institutional benefit was deliberately excluded from the National Insurance Act by the Government (see especially THE HOSPITAL for December 9 and 16, 1911). Mr. Francis is therefore correct in what he says as to this matter of fact. We may point out that Mr. Lloyd George, on introducing the Bill in the House of Commons, laid it down that it would provide free medical relief for every insured person with no taint of charity. We have maintained ever since that the insured have paid for and been State-guaranteed adequate medical and sickness benefit. Mr. Francis evidently does not realise, notwithstanding what he urges, that the Government, through Mr. Masterman, so recently as November 27, in the House of Commons, in reply to questions, stated that "business arrangements can be entered into between hospitals and approved societies in the interest of insured persons under the National Insurance Act." "The societies would arrange, as some do at present, to contribute a certain amount to a hospital in return for a certain number of beds being available for their members." Mr. Masterman thus upholds the principle, and intimates the feasibility under the Act, of the adoption of *pro rata* payments which we have advocated as the way out of the difficulty, so far as the voluntary hospitals are concerned.—ED. THE HOSPITAL.]

### The British Hospitals Association.

*To the Editor of* THE HOSPITAL.

